# A confirmatory study of the Combined Index of Severity of Fibromyalgia (ICAF*): factorial structure, reliability and sensitivity to change

**DOI:** 10.1186/1477-7525-9-39

**Published:** 2011-06-07

**Authors:** Miguel A Vallejo, Javier Rivera, Joaquim Esteve-Vives, Javier Rejas, Group ICAF

**Affiliations:** 1 Facultad de Psicología, Universidad Nacional de Educación a Distancia, Madrid, Spain; 2 Unidad de Reumatología, Instituto Provincial de Rehabilitación, Hospital Universitario Gregorio Marañón, Madrid, Spain; 3 Sección Reumatología, Hospital General Universitari d'Alacant, Alicante, Spain; 4 Departamento de Investigación de Resultados en Salud y Farmacoeconomía, Unidad de Acceso y Relaciones Institucionales, Pfizer España, Alcobendas, Madrid, Spain; 5 ICAF is an acronym in Spanish (Indice Combinado de Afectación de la Fibromialgia) of the English "Combined Index of Severity of Fibromyalgia

## Abstract

**Background:**

Fibromyalgia (FM) is a complex syndrome that affects many aspects of the patients life and it is very difficult to evaluate in clinical practice. A recent study has developed the Combined Index of Severity of Fibromyalgia (ICAF), an instrument that evaluates diverse aspects of FM and offers five indices: emotional, physical, active coping, passive coping and total. The objective of this study is to confirm the structure of the ICAF, check its test-retest reliability, assess its sensitivity to change, and compare the results obtained in a sample of patients with fibromyalgia with another sample of healthy controls.

**Methods:**

A total of 232 patients took part in the study, 228 women and 4 men, with a mean age of 47.73 years of age (SD = 8.61) and a time of disease evolution since diagnosis of 4.28 years (SD = 4.03). The patients from the FM group completed the ICAF. Between one and two weeks later, they again attended the clinic and complete the 59 items on the ICAF (retest) and immediately afterwards they began treatment (according to daily clinical practice criteria). A sample of healthy subjects was also studied as a control group: 110 people were included (106 women and 4 men) with a mean age of 46.01 years of age (SD = 9.35). The study was conducted in Spain.

**Results:**

The results obtained suggest that the four-factor model obtained in the previous study adequately fits the data obtained in this study. The test-retest reliability and internal consistency were all significant and show a high degree of correlation for all the factors as well as in overall score. With the exception of the passive coping factor, all the other scores, including the overall score, were sensitive to change after the therapeutic intervention. The ICAF scores of the patients with fibromyalgia compared with those of the control group were markedly different.

**Conclusions:**

The findings suggest that the ICAF is a valid, reliable, sensitive to change instrument with the added advantage that it offers some additional domains (factors) that provide very valuable information regarding the most delicate aspects of the patient, which must be addressed at the time of treatment in daily clinical practice.

## Background

Fibromyalgia syndrome is a complex syndrome that affects many aspects of the patients life and it is very difficult to evaluate in clinical practice. In addition to the ACR classification criteria [1], which is also the subject of debate [2], it has been defined as the existence of a group of symptoms that includes pain, fatigue, sleep disturbance, morning stiffness, cognitive dysfunction, loss of functional capacity, and emotional aspects such as anxiety, changes in mood or in the way in which the patients face the disease [3].

The mere demarcation of the symptoms indicated has been the object of debate, as have the tools used to evaluate them, due to the enormous quantity of existing instruments [4]. Nonetheless, the symptoms listed have been recognised as relevant when assessing the syndrome; it empirically necessary to establish their relative importance or even determine combined indices that take into account the influence of the symptoms and allow comprehensive indices of the disorder to be obtained [3].

The current efforts being undertaken through Outcome Measures in Rheumatoid Arthritis Clinical Trials (OMERACT) [3] pursue the objective of finding a core data set to evaluate patients with FM in clinical trials. One of the difficulties OMERACT has run up against is due to the fact that some of the symptoms that most concern the patient, such as morning stiffness [5], do not completely concur with those established by OMERACT, and therefore have had to be incorporated to the core data set because they were not initially well-assessed by the investigators.

Furthermore, some of the FM symptoms and domains do not possess good tools that can measure sensitivity to change. Such is the case of quality of life, in which the questionnaires that have been used do not have a good discriminating power [4]. However, a tool is needed in daily clinical practice that allows for a fast, overall evaluation of patients, with an aim to assess the severity of their disease and the most relevant aspects to be dealt with in their treatment. In light of this need, Vallejo et al. [6] has recently developed the Combined Index of Severity of Fibromyalgia (ICAF), an instrument that evaluates the aforementioned symptom areas and offers five indices: emotional, physical, active coping, passive coping and total. The emotional factor stresses the role of emotional aspects such as anxiety and depression; the physical factor evaluates pain, fatigue, sleep quality and functional capacity; the active coping includes positive coping strategies, and passive coping identifies a group of particularly severe affected patients. Global score integrates the four previous factors. This instrument was created by using several other questionnaires that are well-known for their scientific integrity, and the results obtained have allowed for patients to be differentiated based on external criteria, such as medical history, physical functioning tests, occupational situation, etc. In order to understand this research it is highly recommended to read the paper where the ICAF development and instruments used for its validation are described [6].

The objective of this study is to confirm the structure of the ICAF, check its test-retest reliability, assess its sensitivity to change, and compare the results obtained in a sample of patients with fibromyalgia with another sample of healthy controls.

## Methods

### Study design

A longitudinal, open, prospective, comparative, 3-visit study was designed. During the first visit, at baseline, patient demographics and clinical characteristics were collected, and scales included in the study were administered for the first time. After patients completed the evaluation at the second visit (retest), they were prescribed the treatment considered to be most appropriate by the rheumatologist, which consisted of information on any aspects of the disease and individualised prescription of physical exercise and medication, all according to usual daily clinical practice. This treatment did not include any specific psychological treatment or emotional support. A final visit was carried out at 3 months in which the ICAF was applied again. Between these two visits, another optional visit was recommended to adjust the treatment or reinforce compliance, according to individual patient needs. However, at this intermediate visit, no information was recorded and it could be replaced by a telephone call, at the investigator's discretion. The questionnaires used to draft the ICAF were selected from among those commonly used to evaluate the most relevant symptoms of fibromyalgia. The following questionnaires were used: the Fibromyalgia Impact Questionnaire [7-9], the Hospital Anxiety and Depression Scale [10-12], the Brief Pain Inventory [13,14], the Fatigue Assessment Scale [15], the Health Assessment Questionnaire [16,17], the General Health Questionnaire (GHQ-28) [18,19], the Chronic Pain Coping Inventory [20], the Arthritis Self-efficacy Scale [21], and a Sleep Quality Scale where 0 = very good and 10 = very poor. All methods for developing the ICAF have been previously described [6]. The ICAF questionnaire and the scoring system can be found in the Additional File 1.

### Patients, control group and study procedures

A total of 232 patients took part in the study, 228 women and 4 men, with a mean age of 47.73 years of age (SD = 8.61) and a time of disease evolution since diagnosis of 4.28 years (SD = 4.03).

Patient selection was performed by following the same criteria as for the study conducted for the development of the ICAF [6]. The study population was primarily urban and comprised women and men over 18 years of age with a diagnosis of fibromyalgia according to ACR classification criteria [1], recruited consecutively from 15 rheumatology clinics throughout the country. Exclusion criteria included: patients presenting other concomitant diseases with severely impaired physical or functional capacity, rheumatic inflammatory diseases, cardiovascular or pulmonary diseases with poor aerobic capacity, uncontrolled psychiatric diseases, patients involved in litigation processes, and patients included in any other clinical trial.

The patients from the FM group followed the process described below. Once accepted for the study and having signed the informed consent form, they completed the ICAF [6]. Between one and two weeks later, they again attended the clinic to exclusively complete the ICAF (retest) and immediately afterwards they began treatment. The treatments were established according to usual clinical practice criteria, following the recommendations of the SER (Spanish Society of Rheumatology) consensus document [22]. The last visit was performed three months after having started treatment and at this point the patients completed the ICAF again.

A sample of healthy subjects was also studied as a control group. The control group subjects were selected among healthcare personnel and among the companions of those patients who attended the clinic for reasons other than FM, chronic lumbar pain, or other causes of chronic pain. To select the control group, the same exclusion criteria that were used for the FM patient group were used, with the additional criteria that the subjects could not have any chronic disease, clinical symptoms compatible with FM or any type of musculoskeletal pain of any aetiology. When the subjects were accepted, they signed the informed consent form to participate in the study.

The subjects in the control group only filled in the ICAF at the first visit. In this control group, 110 people were included (106 women and 4 men) with a mean age of 46.01 years of age (SD = 9.35). There were no significant differences in terms of age and sex as compared to the FM patient group.

The study protocol was approved by the Independent Ethics Committee of Hospital Universitario Gregorio Marañón (Madrid, Spain). The study was conducted in Spain.

### Statistical analysis

The SPSS statistical package version 17.0 was used throughout.

Confirmatory factor analysis was performed for the results obtained from giving the ICAF and the structure of the questionnaire was put to the test to see whether it fit that obtained previously by Vallejo et al. [6]. To this end, the 4-factor model obtained by said study (emotional, physical, active coping and passive coping) was applied and the AMOS 17 programme was used (AMOS 17 is a component of the SPSS 17.0 statistical package).

The indices of the fit of the model to the data allow for the assessment of whether said model fits the data, keeping in mind the nature of the sample and its variance. In structural equation modelling, there is not a single index or a single set of indices to evaluate the fit of the model. Normally, several indices are calculated that contribute as a group to determining the degree to which the model fits the data [23]. Along these lines, we decided to use the following indices: the relative chi-square (CMIN/DF), the goodness-of-fit index (GFI), Bentler's comparative fit index (CFI) [24], the Tucker-Lewis Index (TLI) [25], and the root mean square error of approximation (RMSEA), following the guidelines by Jackson et al. [26].

The most used and simplest index is the relative chi-square (CMIN/DF). Values below 1 suggest a perfect fit, higher values around 2 or 3 suggest an adequate fit and values close to 5 suggest an inadequate fit [27]. This index is very sensitive to sample size, and therefore other indices must be considered. The GFI behaves in a similar manner as R^2 ^in regression analysis. The CFI varies from 0 to 1. The closer the CFI and GFI values are to 1 [28,29], the better the fit of the model. Values over 0.9 suggest a good fit to the model. The same occurs with the TLI [23]. The RMSEA must have values below 0.8, and the fit is considered to be very good when the values are below 0.5 [30].

The scores obtained in the ICAF were transformed into T scores, as in the previous study [6]. The mean and standard deviation for each scale was taken from that cited in the study mentioned and they can be consulted in the Additional File 1.

To study the test-retest reliability, Spearman's intra-class correlation coefficient (ICC) was determined between the baseline ICAF scores and those obtained later, with an interval of 1-2 weeks. Internal consistency was determined by calculating the Cronbach's alpha coefficient.

To make comparisons between the ICAF scores obtained before and after the therapeutic intervention, a comparison of means was performed using the t-test for related samples. To evaluate the clinical relevant change, standardized response mean (SRM) and the standard error of measurement (SEM) were calculated to determine the change scores when 1 SRM is considered.

Finally, the values obtained in the different ICAF scales of the FM patient group and the control group are shown.

## Results

### Confirmatory factor analysis

The results obtained suggest that the four-factor model obtained in the previous study [6] (Vallejo et al., 2010) adequately fits the data obtained in this study: [X^2 ^(113) = 211.87; CMIN/DF = 1.87; CFI = .91; GFI = .92; TLI = .90; RMSEA = .05]. X^2 ^was statistically significant (p < .001), which suggests that there is a part of the variance that is not explained by the model.

### Test-retest reliability and internal consistency

The ICAF was given to the patients at two different times during the study with an interval of between one to two weeks. The coefficients of correlation obtained between the two sessions can be seen in Table 1. The values of the coefficients were all significant with a value of p < 0.01 and show a high degree of correlation for all the factors as well as in overall score.

**Table 1:** Test-retest reliability and measurement of the internal consistency of the different ICAF factors

Factors	Intraclass correlation coefficient	Cronbach's alpha coefficient
Emotional	0.79	0.88

Physical	0.87	0.77

Active coping	0.87	0.85

Passive coping	0.82	0.77

Overall	0.86	0.85

The internal consistency of each one of the factors according to the Cronbach's alpha coefficient and the ICC can also be seen in Table 1. All the factors show a high degree of internal consistency.

### Sensitivity to change

The comparison between the ICAF scores before and after starting treatment can be observed in Table 2. With the exception of the passive coping factor, all the other scores, including the global score, were sensitive to change after the therapeutic intervention. The SRM shows the responsiveness of the measure to the clinical change. SEM was also calculated to interpret score on Patient Reported Outcome (PRO). This 1 SEM benchmark implies an effect size of 0.20, consistent with Cohen's small-to moderate effect. The mean change score to each component of the ICAF is shown in table 2.

**Table 2:** Sensitivity to change: Comparison of measurements before and after intervention in patients with fibromyalgia

	Mean Pre	Mean Post	**Mean Diff**.	t	SRM	1 SEM Mean change score
Emotional	50.74	47.85	2.89	5.96***	0.48	0.78

Physical Activity	54.27	49.18	5.09	12.12***	0.89	0.62

Active Coping	48.96	50.35	-1.39	-2.37*	0.25	0.53

Passive Coping	51.57	52.65	-1.08	-1.56	0.20	0.84

Total	52.55	48.00	4.55	8.44***	0.66	0.64

N = 22

*p < .05, ***p < .001 SMR: standardized response mean; SEM: standard error of measurement

### Comparison between the patients and controls

**Figure 1:**
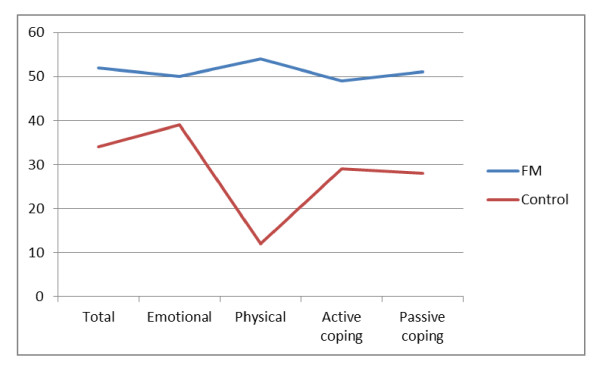
**Graphic representation of mean scores of fibromyalgia patients and controls in the ICAF factors**.

The ICAF scores of the patients with fibromyalgia and those of the control group are shown in Table 3. A graphic representation of mean scores of fibromyalgia patients and controls in the ICAF factors are shown in Figure 1.

**Table 3:** T scores (mean = 50, SD = 10) of fibromyalgia patients and controls

	FM patients, N = 232					Control subjects, N = 110				
	**Total**	**Emot**.	**Phys**.	**Actv.C**.	**Pass.C**.	**Total**	**Emot**.	**Phys**.	**Actv.C**.	**Pass.C**.

Mean	52.55	50.74	54.27	49.11	51.57	34.31	39.78	12.70	29.41	28.21

Minim.	34.05	37.75	18.93	23.38	24.23	22.44	35.61	4.06	23.38	24.23

Maxim.	78.79	68.28	76.30	68.31	72.91	53.46	54.94	45.76	57.49	62.48

Range	44.73	30.53	57.38	44.93	48.68	31.02	19.33	41.70	34.11	38.25

25^th ^Perc.	46.98	45.86	47.61	41.68	43.00	31.34	36.10	6.30	23.38	24.23

50^th^	52.27	49.20	55.44	49.17	52.73	33.14	39.10	10.01	24.21	24.23

75^th^	57.77	54.44	61.40	56.45	59.69	36.03	41.13	16.72	34.40	28.75

Key: Total: general score including all the four factors, Emot.: score for the emotional factor; Phys.: score for the physical factor, Actv.C.: score for the active coping factor, Pass.C.: score for the passive coping factor.

## Discussion

This study used a new sample of fibromyalgia patients to confirm the factorial structure of the ICAF obtained by the previous study [6]. Therefore, it confirms the results obtained as well as the precise reference values to calculate the T scores, as reflected in the Additional File 1. Said Additional File 1 also includes the ICAF, as well as the correction guidelines, in light of the original study data.

The study of the scientific integrity of the instrument was also completed, offering satisfactory data with regard to its test-retest reliability, the internal consistency of the different factors included in the ICAF, as well as its sensitivity to change. The total score of the questionnaire, as well as the scores for the emotional, physical and active coping factors all varied with statistical significance after conventional medical treatment.

There was not a statistically significant change in the passive coping factor. It should be mentioned that in the previous study [6], the greatest score in the passive coping factor was characteristic of poorer physical shape, measured by the 6-minute walk test [31]. Recently, Karsdorp and Vlaeyen [32] found that fibromyalgia patients who refrain from physical activity and seek help (asking for assistance) to control their pain are characterised by greater severity and disability. It is possible that the changes in coping strategies, and particularly the passive strategies, require longer periods of intervention and evaluation. The pain-avoidance factor (passive pain-coping) is particularly relevant to determine a risk of longer aggravated distress in fibromyalgia [33]. It is reasonable that the change score for passive coping is higher (0.8391) than in the other factors. This implies that the minimal score change (1 SEM) in this factor should also be higher than in the others factors and corresponds well with the level of distress associated with passive coping [33]. This should be dealt with through therapeutic intervention studies that have a longer duration, using psychological treatment and physical exercise.

The results obtained in passive coping must be considered in relation with gender. In FM studies female percentages are about 95% or more [32,33], which indicates that there is an influence of specific gender differences in pain perception, specifically in coping with pain. Women report more pain than men [34], and men use more active coping than women [35]. It is likely that female FM patients may have more difficulties to reduce passive coping and to increase active coping.

The ICAF scores in patients and controls show large differences, both regarding the overall score and each of the four factors, which emphasises the specificity of ICAF for fibromyalgia patients. This is especially pronounced both active and passive coping factors. The control subjects do not have symptoms that interfere with their lives, namely pain, and thus it clearly makes no sense to discuss coping strategies.

The differences are still more evident on the physical scale, in which the mean patient score is four times that obtained with the controls (see Table 3).

All these data show the instrument's capacity to differentiate between the two population groups.

Therefore, the ICAF has a set of domains relevant to fibromyalgia and it largely responds to the need mentioned by several authors: to have an extensive set of measures for the syndrome that can be used in both clinical trials [3] and in daily clinical practice. The ICAF was developed and compared to physical functionality tests, encompassing the domains in which there was the greatest consensus at OMERACT 9 [3].

This instrument also includes the evaluation of emotional aspects such as depression and anxiety. The latter is known as a domain of interest for research, as distress may contribute to increasing the importance of the painful points characteristic of the ACR classification.

One of the inconveniences of the ICAF may be its length, given that it includes 59 items, compared to most of the tools used for FM patients. However, if we consider that to obtain the same information collected by the ICAF, several different tools must be used together, adding up into many more items, we can conclude that the ICAF saves a considerable amount of time for both the doctor and the patient. The average time that the patient needs to fill the ICAF is 15 minutes.

The ICAF shows well balanced information about the main areas of severity in FM. Some scales have a small but clinically significant relevance, such as passive coping, while the remaining scales, as well as global score, are very useful for understanding the clinical situation of the patient and for starting up with treatment.

In relation with the length of the ICAF, it is possible that future studies may reduce the number of items preserving the information offered.

The fact that this study was conducted in Spain may be a limitation to generalize ICAF to other patients in different countries. This limitation may affect the ICAF structure but not the items because they were selected from well-known instruments validated in most countries [6].

## Conclusions

The findings suggest that the ICAF is a valid, reliable, sensitive to change instrument with the added advantage that it offers some additional domains (factors) that provide very valuable information regarding the most delicate aspects of the patient, which must be addressed at the time of treatment.

The areas of evaluation of the ICAF also coincide, in general, with those proposed by Wolfe et al. [2], and have also been developed with two data sources: those coming from the patient and those from the medical examination.

## List of abbreviations used

ACR: American College of Rheumatology; CFI: Comparative Fit Index; FM: Fibromyalgia; CMIN/DF: Relative Chi-square; GHQ-28: General Health Questionnaire 28; GFI: Goodness-of-Fit Index; ICAF: acronym in Spanish (Indice Combinado de Afectación de la Fibromialgia) of the English "Combined Index of Severity of Fibromyalgia"; ICC: Intraclass Correlation Coefficient; OMERACT: Outcome Measures in Rheumatoid Arthritis Clinical Trials; PRO: Patient Reported Outome; RMSEA: Root Mean Square Error of Approximation; SEM: Standard Error of Measurement; SER: In Spanish (Sociedad Española de Reumatología) of the English "Spanish Society of Rheumatology"; SRM: Standardized Response Mean; TLI: Tucker-Lewis Index.

## Competing interests

This research was supported by a grant of Pfizer Laboratory and Fondo de Investigaciones Sanitarias (FIS) PI 07/0202.

There are no financial or other conflicts of interest of which we are aware.

## Authors' contributions

JRivera, MAV and JEV conceived the study. MAV designed the study and perform the statistical analysis. JRivera and JRejas participated in the statistical analysis and interpretation of the data. These authors revised the data obtained and draft the manuscript. MAV coordinated the analysis, results and discussion. All the authors read and approved the final manuscript.

ICAF Group authors contributed only in the data acquisition.

## Supplementary Material

Additional file 1 Appendix Click here for file
