# Supplementary material for: A confirmatory study of the Combined Index of Severity of Fibromyalgia (ICAF*): factorial structure, reliability and sensitivity to change
Source: Health Qual Life Outcomes. 2011 Jun 7;9:39. doi: 10.1186/1477-7525-9-39 (PMC3127741; doi:10.1186/1477-7525-9-39)
Supplement: Additional file 1 — Appendix [file 1477-7525-9-39-S1.doc]

**ICAF**

**INSTRUCTIONS**

We would like to know how the symptoms of your disease were **DURING THE LAST WEEK.** Please circle only one response for each question.

### PAIN SEVERITY

1.- Please rate your pain by circling the one number that best describes your pain at its **LEAST** in the **LAST WEEK.**

          

0 1 2 3 4 5 6 7 8 9 10

No pain Pain as bad as

you can imagine

2.- Please rate your pain by circling the one number that best describes your pain on the **AVERAGE.**

          

0 1 2 3 4 5 6 7 8 9 10

No pain Pain as bad as

you can imagine

| **SCORE 1** |  |
| --- | --- |
| (Items 1+2) |  |
| Range 0-20 |  |

### SLEEP QUALITY

3.- Please circle the number that best describes **HOW YOU SLEPT LAST WEEK**.

          

0 1 2 3 4 5 6 7 8 9 10

Very well Very bad

Very well Very bad

| **SCORE 2** |  |
| --- | --- |
| (item 3) |  |
| Range 0-10 |  |

### IMPACT

Please circle the number that best describes how you **FELT OVERALL** for the **PAST WEEK**

4.- When you worked, how much did pain or other symptoms of your fibromyalgia **INTERFERE** with your ability to do **YOUR WORK, INCLUDING HOUSEWORK?**

          

0 1 2 3 4 5 6 7 8 9 10

No problem with work Great difficulty with work

5.-How **TIRED** have you been ?

          

0 1 2 3 4 5 6 7 8 9 10

No tiredness Very tired

6.-How have you felt when you **GOT UP IN THE MORNING** ?

          

0 1 2 3 4 5 6 7 8 9 10

Awoke well rested Awoke very tired

| **SCORE 3** |  |
| --- | --- |
| (Items 4+5+6) |  |
| Range 0-30 |  |

**FATIGUE**

Please circle the number that best describes how you usually **FELT for PAST WEEK.**

|  | Never | Some  times | Regularly | Often | Always |
| --- | --- | --- | --- | --- | --- |
| 7 - I am bothered by fatigue | 0 | 1 | 2 | 3 | 4 |
| 8 - I get tired very quickly | 0 | 1 | 2 | 3 | 4 |
| 9 - I don’t do much during the day | 0 | 1 | 2 | 3 | 4 |
| 10 - Physically, I feel exhausted | 0 | 1 | 2 | 3 | 4 |
| 11 - I have problems starting things | 0 | 1 | 2 | 3 | 4 |

| **SCORE 4** |  |
| --- | --- |
| (Items 7-11) |  |
| Range 0-20 |  |

**PHYSICAL FUNCTION**

Please check the one response which best describes **YOUR USUAL ABILITIES** over the **PAST WEEK**

| **Are you able to…** | Without ANY difficulty | With SOME difficulty | With MUCH difficulty | UNABLE to do |
| --- | --- | --- | --- | --- |
| 12 - Dress yourself, including tying shoelaces and doing buttons? | 0 | 1 | 2 | 3 |
| 13 - Wash and dry your entire body? | 0 | 1 | 2 | 3 |
| 14 - Reach and get down a 5-pound object (such as a bag of sugar) from just above your head? | 0 | 1 | 2 | 3 |
| 15 - Get in and out of a car? | 0 | 1 | 2 | 3 |
| 16 – Do chores such as vacuuming or yardwork? | 0 | 1 | 2 | 3 |

| **SCORE 5** |  |
| --- | --- |
| (Items 12-16) |  |
| Range 0-15 |  |

## ANXIETY AND DEPRESSION

Read each item and mark the reply which comes closest to how you have been FEELING in the PAST WEEK**.**

| **17 - I feel tense or 'wound up':** | | |
| --- | --- | --- |
| (3) |  | Most of the time |
| (2) |  | A lot of the time |
| (1) |  | From time to time, occasionally |
| (0) |  | Not at all |
|  | | |
| **18 - I still enjoy the things I used to enjoy:** | | |
| (0) |  | Definitely as much |
| (1) |  | Not quite so much |
| (2) |  | Only a little |
| (3) |  | Hardly at all |
|  |  |  |
| **19- I can laugh and see the funny side of things:** | | |
| (0) |  | As much as I always could |
| (1) |  | Not quite so much now |
| (2) |  | Definitely not so much now |
| (3) |  | Not at all |
|  |  |  |
| **20 - Worrying thoughts go through my mind:** | | |
| (3) |  | A great deal of the time |
| (2) |  | A lot of the time |
| (1) |  | From time to time, but not too often |
| (0) |  | Only occasionally |
|  |  |  |
| **21 - I look forward with enjoyment to things:** | | |
| (0) |  | As much as I ever did |
| (1) |  | Rather less than I used to |
| (2) |  | Definitely less than I used to |
| (3) |  | Hardly at all |
|  |  |  |
| **22 - I get sudden feelings of panic:** | | |
| (3) |  | Very often indeed |
| (2) |  | Quite often |
| (1) |  | Not very often |
| (0) |  | Not at all |

| **SCORE 6** |  |
| --- | --- |
| (Items 17-22) |  |
| Range 0-18 |  |

**GENERAL HEALTH**

We should like to know if you have had any medical complaints and how your health has been in general, over the past week.

During the **PAST WEEK** **HAVE YOU**…

| **23 - Felt constantly under strain?** | | |
| --- | --- | --- |
| (0) |  | Not at all |
| (1) |  | No more than usual |
| (2) |  | Rather more than usual |
| (3) |  | Much more than usual |
|  | | |
| **24 - Been getting edgy and bad-tempered?** | | |
| (0) |  | Not at all |
| (1) |  | No more than usual |
| (2) |  | Rather more than usual |
| (3) |  | Much more than usual |
|  |  |  |
| **25 - Found everything getting on top of you?** | | |
| (0) |  | Not at all |
| (1) |  | No more than usual |
| (2) |  | Rather more than usual |
| (3) |  | Much more than usual |
|  |  |  |
| **26 - Been feeling nervous and strung-up all the time?** | | |
| (0) |  | Not at all |
| (1) |  | No more than usual |
| (2) |  | Rather more than usual |
| (3) |  | Much more than usual |
|  |  |  |
| **27 - Felt on the whole you were doing things well?** | | |
| (0) |  | Better than usual |
| (1) |  | About the same |
| (2) |  | Less well than usual |
| (3) |  | Much less well |
|  |  |  |
| **28 - Been satisfied with the way you’ve carried out your task?** | | |
| (0) |  | More satisfied |
| (1) |  | About same as usual |
| (2) |  | Less satisfied than usual |
| (3) |  | Much less satisfied |
|  |  |  |
| **29 - Been able to enjoy your normal day-to-day activities?** | | |
| (0) |  | More so than usual |
| (1) |  | Same than usual |
| (2) |  | Less so than usual |
| (3) |  | Much less than usual |
|  |  |  |
| **30 - Felt that life isn’t worth living?** | | |
| (0) |  | Not at all |
| (1) |  | No more than usual |
| (2) |  | Rather more than usual |
| (3) |  | Much more than usual |
|  |  |  |
| **31 - Thought of the possibility that you might make away with yourself?** | | |
| (0) |  | Definitely not |
| (1) |  | I don’t think so |
| (2) |  | Has crossed my mind |
| (3) |  | Definitely have |
|  |  |  |
| **32 - Found yourself wishing you were dead and away from it all?** | | |
| (0) |  | Not at all |
| (1) |  | No more than usual |
| (2) |  | Rather more than usual |
| (3) |  | Much more than usual |
|  |  |  |
| **33 -** **Found that the idea of taking your own life kept coming into your mind?** | | |
| (0) |  | Definitely not |
| (1) |  | I don’t think so |
| (2) |  | Has crossed my mind |
| (3) |  | Definitely have |

| **SCORE 7** |  |
| --- | --- |
| (Items 23-33) |  |
| Range 0-33 |  |

**COPING STRATEGIES**

During the **PAST WEEK,** how many days did you use each of the following at least once in the day to cope with your pain? (Please, indicate the number of days you used each strategy for pain, whether or not you were experiencing pain at the time.

|  | | **Number of days** | | | | | | | |
| --- | --- | --- | --- | --- | --- | --- | --- | --- | --- |
|  |  | **0** | **1** | **2** | **3** | **4** | **5** | **6** | **7** |
| 34 - | Imagined a calming or distracting image to help me relax |  |  |  |  |  |  |  |  |
| 35 - | Ignored the pain |  |  |  |  |  |  |  |  |
| 36 - | Asked someone to do something for me |  |  |  |  |  |  |  |  |
| 37 - | Focussed on relaxing my muscles |  |  |  |  |  |  |  |  |
| 38 - | Held on to something when getting up or sitting down |  |  |  |  |  |  |  |  |
| 39 - | Told myself things will get better |  |  |  |  |  |  |  |  |
| 40 - | I got support from a family member |  |  |  |  |  |  |  |  |
| 41 - | Thought about all the good things I have |  |  |  |  |  |  |  |  |
| 42 - | Asked for help in carrying, lifting or pushing something |  |  |  |  |  |  |  |  |
| 43 - | Told myself my pain will get better |  |  |  |  |  |  |  |  |
| 44 - | Avoided putting weight on feet or legs |  |  |  |  |  |  |  |  |
| 45 - | I didn’t let the pain interfere with my activities |  |  |  |  |  |  |  |  |
| 46 - | Limited my walking because of pain |  |  |  |  |  |  |  |  |
| 47 - | Just didn’t pay attention to the pain |  |  |  |  |  |  |  |  |
| 48 - | Talked to a friend or family member for support |  |  |  |  |  |  |  |  |
| 49 - | I just kept going |  |  |  |  |  |  |  |  |
| 50 - | Lay down on a bed |  |  |  |  |  |  |  |  |
| 51 - | Reminded myself about things that I have going for me such as intelligence, good looks, and good friends |  |  |  |  |  |  |  |  |
| 52 - | Got together with a family member |  |  |  |  |  |  |  |  |
| 53 - | Used deep, slow breathing to relax |  |  |  |  |  |  |  |  |
| 54 - | Went into a room by myself to rest |  |  |  |  |  |  |  |  |
| 55 - | Did not let the pain affect what I was doing |  |  |  |  |  |  |  |  |

| **SCORE 8** |  |
| --- | --- |
| (Items in blank |  |
| 34+35+37+39+41+43+45+47+49+51+53+55) |  |
| Range 0-84 |  |

| **SCORE 9** |  |
| --- | --- |
| (Items in grey |  |
| 36+38+40+42+44+46+48+50+52+54) |  |
| Range 0-70 |  |

**SELF EFFICACY**

We should like to know your opinion about **YOUR ABILITY TO CONTROL FIBROMYALGIA SYMPTOMS**.

Please circle the number that corresponds to how certain you are that you can do the following tasks regularly at the present time.

56 - **How certain are you that you can decrease your pain quite a bit?**

          

0 1 2 3 4 5 6 7 8 9 10

Very uncertain Very certain

57 - **How certain are you that you can keep your fibromyalgia pain from interfering with your sleep?**

          

0 1 2 3 4 5 6 7 8 9 10

Very uncertain Very certain

58 - **How certain are you that you can do something to help yourself feel better if you are feeling blue?**

          

0 1 2 3 4 5 6 7 8 9 10

Very uncertain Very certain

59 - **As compared with other people with fibromyalgia like yours, how certain are you that you can manage pain during your daily activities?**

          

0 1 2 3 4 5 6 7 8 9 10

Very uncertain Very certain

| **SCORE 10** |  |
| --- | --- |
| (Items 56-59) |  |
| Range 0-40 |  |
|  |  |

**ICAF SCORING SHEET**

| Physical | | Active coping | |  |
| --- | --- | --- | --- | --- |
| **Direct score**  (1+2+3+4+5) |  | **Direct score**  (8+10) |  |  |
| Z score^1^  (Direct - 61.7) / 13.43 |  | Z score^1^  (Direct - 63.99) / 24.04 |  |  |
| T score^2^  (z * 10) + 50 |  | T score^2^  (z * 10) + 50 |  |  |
| Emotional | | Passive coping | |  |
| **Direct score**  (6+7) |  | **Direct score**  (9) |  |  |
| Z score^1^  (Direct - 23.84) / 10.48 |  | Z score^1^  (Direct - 37.06) / 14.38 |  |  |
| T score^2^  (z * 10) + 50 |  | T score^2^  (z * 10) + 50 |  |  |
|  |  |  |  |  |

For ICAF TOTAL calculation, **use direct scores** in the following formula:

| Physical  *  0,23 |  | Emotional  *  0,53 |  | Passive coping  *  0,1 |  | Active coping  *  0,14 |  | **Direct score** |
| --- | --- | --- | --- | --- | --- | --- | --- | --- |
|  | **+** |  | **+** |  | **−** |  | **=** |  |

ICAF

| Z score^1^  (Direct – 21.38 ) / 9.8 |  |  | T score^2^  (z * 10) + 50 |  |
| --- | --- | --- | --- | --- |

Z scores are based in a previous study with 301 patients with fibromialgia (Vallejo MA, Rivera J, Esteve-Vives J, Group ICAF: Development of a self-reporting tool to obtain a Combined Index of Severity of Fibromyalgia (ICAF). *Health Qual Life Outcomes 2010,8*:2.

<http://www.hqlo.com/content/8/1/2>)

^2^ T scores have a media = 50, and a standard deviation = 10.
